# Activation of Sphingomyelinase-Ceramide-Pathway in COVID-19 Purposes Its Inhibition for Therapeutic Strategies

**DOI:** 10.3389/fimmu.2021.784989

**Published:** 2021-12-20

**Authors:** Murad Abusukhun, Martin S. Winkler, Stefan Pöhlmann, Onnen Moerer, Konrad Meissner, Björn Tampe, Heike Hofmann-Winkler, Michael Bauer, Markus H. Gräler, Ralf A. Claus

**Affiliations:** ^1^ Department of Anesthesiology and Intensive Care Medicine, Jena University Hospital, Jena, Germany; ^2^ Center for Molecular Biomedicine (CMB), Jena University Hospital, Jena, Germany; ^3^ Department of Anesthesiology, Emergency and Intensive Care Medicine, University of Göttingen, Göttingen, Germany; ^4^ Infection Biology Unit, German Primate Center-Leibniz Institute for Primate Research, Göttingen, Germany; ^5^ Faculty of Biology and Psychology, University Göttingen, Göttingen, Germany; ^6^ Department of Nephrology, University of Göttingen, Göttingen, Germany; ^7^ Center for Sepsis Control and Care (CSCC), Jena University Hospital, Jena, Germany

**Keywords:** molecular biology of critical care, ceramide, organ failure, sphingomyelinase, FIASMA, drug repurposing, molecular markers

## Abstract

Effective treatment strategies for severe coronavirus disease (COVID-19) remain scarce. Hydrolysis of membrane-embedded, inert sphingomyelin by stress responsive sphingomyelinases is a hallmark of adaptive responses and cellular repair. As demonstrated in experimental and observational clinical studies, the transient and stress-triggered release of a sphingomyelinase, SMPD1, into circulation and subsequent ceramide generation provides a promising target for FDA-approved drugs. Here, we report the activation of sphingomyelinase-ceramide pathway in 23 intensive care patients with severe COVID-19. We observed an increase of circulating activity of sphingomyelinase with subsequent derangement of sphingolipids in serum lipoproteins and from red blood cells (RBC). Consistent with increased ceramide levels derived from the inert membrane constituent sphingomyelin, increased activity of acid sphingomyelinase (ASM) accurately distinguished the patient cohort undergoing intensive care from healthy controls. Positive correlational analyses with biomarkers of severe clinical phenotype support the concept of an essential pathophysiological role of ASM in the course of SARS-CoV-2 infection as well as of a promising role for functional inhibition with anti-inflammatory agents in SARS-CoV-2 infection as also proposed in independent observational studies. We conclude that large-sized multicenter, interventional trials are now needed to evaluate the potential benefit of functional inhibition of this sphingomyelinase in critically ill patients with COVID-19.

## Introduction

The rampant spreading of the novel severe acute respiratory virus-2 (SARS-CoV-2) with an estimated global infection rate of 10% causing coronavirus disease 2019 (COVID-19) has resulted in an unprecedented pandemic crisis of health care systems worldwide (1). COVID-19 is a new disease entity and severe cases have a high mortality due to the fact that SARS-CoV-2 is systemic and potentially affects all organs (2–4). The clinical course of COVID-19 is highly variable, which is also reflected by a wide range of symptoms, such as an asymptomatic course up to self-induced, over-exuberant inflammation and acute respiratory distress syndrome (ARDS) with multiple organ dysfunction and death (5). The underlying reasons for heterogeneous clinical courses are not yet completely understood, but current data suggest that a plethora of epidemiological factors such as age, gender or pre-existing conditions and its medical treatment combined with genetic susceptibility and as well as virus associated factors such as viral load contribute to the outcome of patients (6–10). Beside continuously increasing vaccination rates in developed countries, therapeutic strategies targeting the immune response, the cytokine release, and endothelial cell barrier integrity are under development in larger clinical trials (11). However, a majority of trials is based on vague assumptions regarding the pathophysiological mechanisms of COVID-19, and the development of causative treatment strategies is hampered due to lack of disease-specific knowledge (12–14).

As an adaptive response mechanism towards cellular damage, the conserved stress responsive enzyme acid sphingomyelinase (ASM, systematically SMPD1) is released into circulation and is held responsible for the rapid and transient formation of ceramide, which is a highly bioactive lipid mediator involved in cellular activation, damage repair, pathogen penetration, danger signaling, maintenance of endothelial integrity and induction of apoptosis (15–17). ASM is released from lysosomes to the outer leaflet of cellular membranes, which are composed from high amounts of sphingomyelin functioning as the embedded substrate to the enzyme. ASM occurs at a low level under physiological conditions, however, the release of the enzyme as a consequence of lysosomal exocytosis in response to stress (18) is suggested to be mostly relevant as a major source of circulating activity in the course of sepsis (19, 20) and pneumonia (21). Activity levels of the enzyme are discriminative for prediction of unfavorable outcome in patients with polymicrobial sepsis (19, 20, 22).

Recently*, in-vitro* observations showed that ASM is also activated upon infection of epithelial cells with SARS-CoV-2. Neutralization or inhibition of subsequent ceramide generation is able to prevent both entry and propagation of SARS-CoV-2 as well as of pseudoviral particles presenting SARS-CoV-2 spike protein, a *bona fide* system mimicking SARS-CoV-2 infection (23–25).

There is accumulating evidence that a substantial number of critically ill COVID-19 patients frequently exhibit viral RNAemia accompanied with a dysregulated immune response (26) fulfilling SEPSIS-III criteria (27, 28) with hyperinflammation manifesting as a cytokine storm or as cytokine release syndrome, which in turn contributes to the high mortality rates (29, 30). From a molecular perspective, inhibition and inactivation of ASM provide anti-inflammatory properties by a decrease of tumor necrosis factor (TNF) α and interleukin (IL)-6 as well as - in a reflective manner - of IL-10 (31, 32), which are all highly correlated with morbidity and mortality rate of COVID-19 (33, 34).

In this inter-relationship, we hypothesize that activation of sphingomyelinase-ceramide pathway might play a crucial role in the pathogenesis of COVID-19: (1) epithelial tissue damage to infection and subsequent repair might result in the serum appearance of ASM in critically ill patients, of which the activity is associated with severity markers, as well as (2) in rather long-lasting compartments (lipoproteins and erythrocytic membranes) the change of activity is mirrored by an increase of ceramides, reflecting the deteriorated status of the patients.

## Methods

Twenty-three COVID-19 patients treated in the intensive care unit (ICU) of the Department of Anesthesiology at Göttingen University Medical Centre (UMG) from March 2020 to May 2020 were enrolled into this study. The local ethics board at UMG approved inclusion of all ICU patients (reference 15/4/19Ü). Informed consent has been obtained from patients or their legal representatives from all study participants prior to inclusion. Data from this report are partially achieved from a re-analysis of samples firstly reported in 2021^1^.

For clinical evaluation, SOFA scores were calculated on admission according to the published guidelines (35). Within the first 24h after inclusion, serum samples were taken to measure ceramide profile and circulating sphingomyelinase activity. Leukocyte-free RBC were harvested from separate samples by density gradient centrifugation, washed and stored in plasma-free conditions at 4°C.

Ceramide measurements were performed according to an established protocol using liquid chromatography coupled to triple-quadrupole mass spectrometry (LC-MS/MS) (36). From serum samples as well as pelleted RBC, proteins were precipitated by addition of methanol supplemented with appropriate internal standard solutions. Following separation of supernatant, evaporation and resolubilization, detection was performed with the QTrap triple-quadrupole mass spectrometer (Sciex, Darmstadt, Germany) interfaced with the 1100 series chromatograph and the Hitachi Elite LaChrom column oven and autosampler. Positive electrospray ionization (ESI) LC/MS/MS analysis was used for detection of sphingomyelins, positive atmospheric pressure chemical ionization (APCI) for ceramides. Standard curves were generated by adding increasing concentrations of ceramide up to 100 pmol of the internal standard C15-ceramide. Linearity of the standard curves and correlation coefficients were obtained by linear regression analyses (r^2^ > 0.99). Data analyses were performed using Analyst 1.6 (Sciex).

For determination of circulating sphingomyelinase activity, serum samples were dissolved with reaction buffer and supplemented with substrate solution. After incubation, extraction, evaporation and resolubilization, detection of C17-Cer (d18:1) for the conversion from C17-SM (d18:1) was performed with the API2000 triple-quadrupole mass spectrometer.

For statistical analysis, primary variables were ceramide isoforms (n=6) either in serum or RBC, and C17-Cer(d18:1) as product of sphingomyelinase activity. Outliers were identified according to the ROUT method with the desired maximum false discovery rate Q set to 1% (37). Differences between two groups were tested for significance using the unpaired Student’s t-test with nonparametric correction using the Mann-Whitney U-test. A p-value <0.05 was considered to be significant. Statistical analyses were performed using Graph Pad Prism 7.0a, April 2016 (La Jolla, CA, USA). Graphical illustration of variations and principal component analyses were performed using metaboanalyst platform [5.0 (38)].

Additional detail on the methods for making these measurements is provided in an online data supplement.

## Results

### Study Population

In order to investigate the ASM/ceramide signaling in SARS-CoV-2 infection, blood samples of 23 patients and six healthy volunteers were collected. Clinical data in **Table 1** summarize socio-demographic and selected clinical characteristics of the study group (day 1 of patients at intensive care unit due to COVID-19) and healthy controls. All patients received standard treatment by the time of admission, which did not include specific anti-viral or general anti-inflammatory drugs such as dexamethasone, because it was not standard of care at time of inclusion. The clinical severity assessment by use of sequential organ failure assessment (SOFA) and the simplified acute physiology score (SAPS II) were 9 (5-11, SOFA) and 41 (34-49, SAPS II) at enrollment. Predicted mortality rate of COVID-19 patients was matching that observed at ICU (26.6. vs. 30%). Parameters of healthy controls were all found in normal range. In contrast, a significant reduction in hemoglobin, hematocrit, RBC count, mean corpuscular hemoglobin concentration (MCHC), serum albumin, and lipoproteins were observed in COVID-19 patients.

**Table 1:** Socio-demographic and clinical parameters of patients and healthy controls.

Parameter	Normal range	COVID-19	Healthy controls	p-value
n		23	5	
Hemoglobin, g/dL	13.5-17.5	10.5 (8.8-12.5)	15.6 (14.0-15.9)	<0.001
Hematocrit, %	39-51	33 (27-37)	46 (41-46)	<0.001
Red blood cells, 10^6^/µL	4.4-5.9	3.79 (3.17-4.26)	5.1 (4,3-5.3)	<0.01
MCV, fL	81-95	87 (77-93)	91 (86-95)	0.219
MCH, pg	26-32	28 (24-31)	31 (29-32)	0.104
MCHC, g/dL	32-36	32.6 (31.5-33.3)	34 (33.8-34.2)	<0.001
Platelets, 10^3^/µL	150-350	195 (131-327)	285 (245-309)	0.343
Leucocytes, 10^3^/µL	4.0-11.0	11.1 (5.6-13.6)	5.6 (5.5-7.2)	0.110
Lymphocyte, %	20-45	9.5 (5.9-25.1)	37.0 (33.0-47.5)	<0.01
Monocytes, %	3-13	7.1 (4.2-8.2)	8.0 (7.0-9.5)	0.148
Eosinophiles, %	≤8	1.0 (0.35-1.5)	3.0 (2.5-3.0)	<0.05
Basophiles, %	≤2	0.40 (0.15-1.0)	0.05 (0.03-0.08)	N/D
Neutrophiles, %	40-76	69 (56-82)	52 (41-53)	<0.05
Serum albumin, g/dL	3.4-5.5	2.1 (1.7-2.3)	4.7 (4.6-4.7)	<0.001
Cholesterol, mg/dL	≤200	120 (96-143)	225 (178-246)	<0.001
Triglycerides, mg/dl	≤150	135 (102-243)	100 (94-141)	0.182
LDL mg/dL	≤115	71 (50-85)	150 (124-183)	<0.001
HDL mg/dL	>40	18 (11-32)	54 (44-64)	<0.001
**Parameter**	**Normal range**	**COVID-19**		
SARS-CoV-2, GE/reaction	N/A	468 (28-22011)		
Age, y	N/A	69 (66-75)		
Died, n (%)	N/A	7 (30%)		
ICU days	N/A	16 (7-24)		
ECMO, n (%)	N/A	3 (13%)		
SAPS II	N/A	41 (34-49)		
Predicted mortality, %	N/A	26.6 (15.3-43.8)		
SOFA	N/A	9 (5-11)		
FiO_2_	N/A	0.50 (0.37-0.75)		
paO_2_, mmHg	65-105	73 (66-88)		
paCO_2_, mmHg	36-42	44 (36-62)		
paO_2_/FiO_2_	N/A	133 (86-217)		
SpO_2_	94-98	96 (95-98)		
pH	7.34-7.45	7.42 (7.34-7.46)		
Temperature, °C	N/A	38.0 (37.0-38.5)		
D-Dimers, mg/L	<0.5	1.34 (0.72-3.52)		
C-reactive protein, mg/dL	≤5	118.7 (29.7-186.6)		
Ferritine, µg/L	22-275	732 (163-1337)		
Procalcitonin, µg/L	<0.07	0.50 (0.15-1.30)		
Interleukin-6, pg/mL	<7	75.4 (45.1-514.1)		
Lactate, mmol/L	≤1.8	1.1 (0.6-1.6)		

GE, genome equivalent; N/A, not available.

### Metabolite Detection

**Figure 1:**
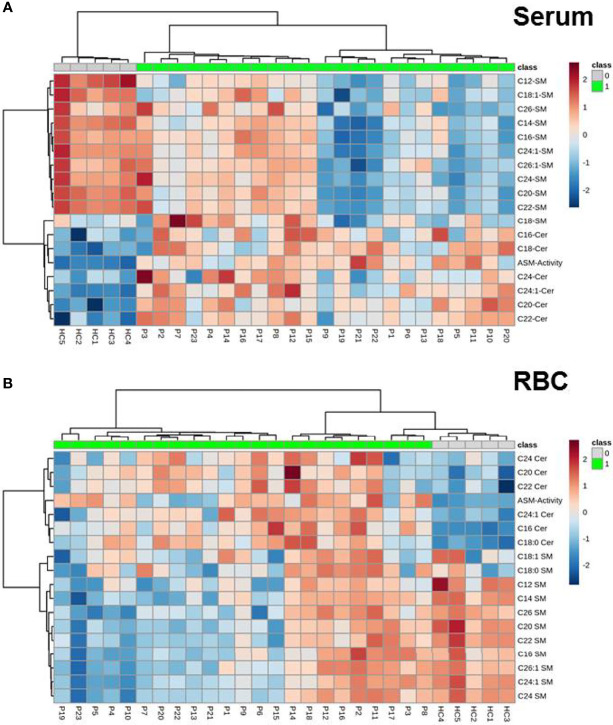
Heatmap of spingolipids (SM and ceramides) as well as ASM activity either in **(A)** serum and **(B)** RBC. Shown are hierarchical cluster analyses from data obtained by monitoring the profile of sphingomyelins and ceramides as well as ASM activity (measured in serum samples). Specimen of sphingolipids are given in rows, individual patients samples in columns comparing patients (P1 –P23, marked in green) and healthy controls (HC 1-6, gray). Color code: increase in sphingolipid content is given in red, decrease in blue. Distance measure is given in euclidian manner.

First, we analyzed the pattern of sphingomyelin in lipid extracts of red blood cells and serum samples. Analysis of serum samples revealed that sphingomyelin levels in circulating lipoproteins were decreased in COVID-19 patients as compared to healthy controls (**Figure 1A**). Since host response and hypoxia have profound effects on RBCs’ morphology, rheology and functional activity (39), we thus sought to assess the composition of this cellular subpopulation. Similar results were obtained in RBCs, a reduction of sphingolipid content in these cells supports the concept of a deranged sphingomyelin balance in RBC-membranes in COVID-19 (**Figure 1B**). Next, we analyzed metabolites and degradation products of sphingomyelin. Therefore, we analyzed ceramide species with a naturally occurring sphingoid backbone (d18:1), but without any modification (n=11) with a chain length in a range between 12 and 26 carbon atoms and in part with an unsaturated double bond (*i.e.* 12:0, 14:0, 16:0, 18:0, 18:1, 20:0, 22:0, 24:0, 24:1, 26:0 and 26:1). Thereof, six ceramide species were found above lower limit of detection (LOD) in > 75% of all samples, which were included in subsequent analyses. Our results - presenting a specific disease pattern of concurring ceramide specimen - are in line with information from previously published cohorts (21). In serum as well as in RBC, Cer was found to be increased in COVID-19 patients and this increase was paralleled by a decrease of SM (**Figure 1**).

### Changes in Ceramide Profile in Patients With COVID-19

**Figure 2:**
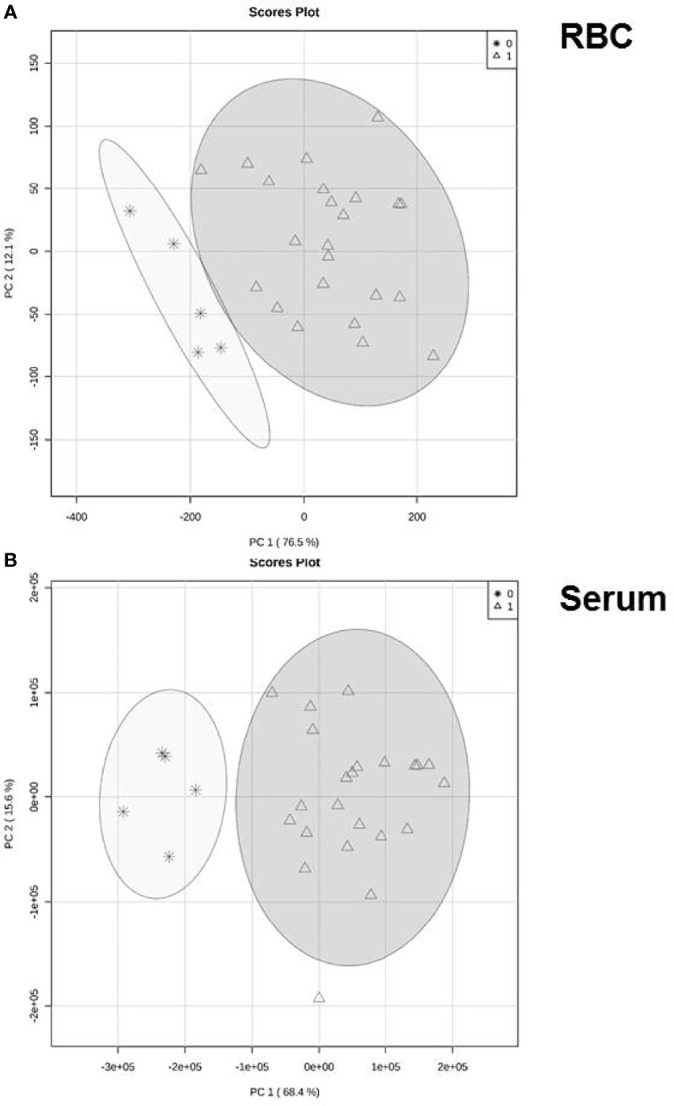
Reprogramming of ceramide generation. Variation of ceramide profile either in RBC **(A)** or serum **(B)** in patients with COVID-19 compared to healthy volunteers. Principal component analysis (PCA) based on concentration profile of six ceramide specimen (16:0, 18:0, 20:0, 22:0, 24:0, 24:1) that passed the quality screen. Each circle represents the centroid of all samples in the representative group at day 1 of intensive care of COVID-19 patients). Healthy controls are given as asterisks, patients in triangles.

Principal component analysis was then used to test whether differences in the ceramide profile reflected the clinical diagnosis (**Figure 2**). In both compartments, serum samples as well as extracts from RBC, values clustered distinctly between patients and healthy controls. For RBC the first and second principal components parameters were 76.5 and 12.1% (**Figure 2A**), for serum 68.4 and 15.6% (**Figure 2B**), respectively.

**Figure 3:**
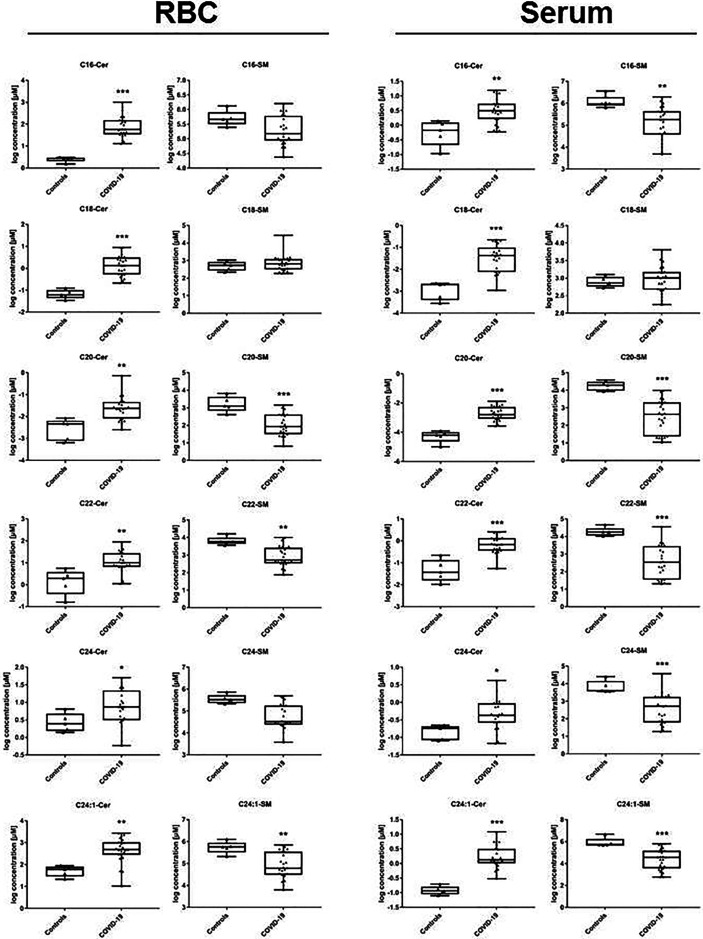
Ceramide profile in lipid extracts obtained from RBC and from serum samples. In patients with COVID-19, ceramide synthesis is increased in all specimen passing quality screen in RBC, and nearly in all investigated in serum (one exception: 24:0). Total amount of ceramide specimen is differing as expected between serum and RBC, absolute values are given in **Table 3**. Statistical analysis was performed using Mann-Whitney U-Test and p-values <0.05 were considered to be significant. COVID-19 *vs.* healthy controls: *p < 0.05; **p < 0.01; ***p < 0.001. Exact parameters indicating statistical difference area also given in **Table 2**.

Concentration levels of the majority of ceramide specimens were significantly changed in patients with COVID-19 with respect to controls, either in serum or RBC. The absolute amount of investigated specimen varied with respect to expected baseline levels (**Figure 3**). An overview of resulting p-values comparing the groups is given in **Table 2**. Median values of individual ceramide specimen including interquartile range (Q1/Q3) are given in **Table 3** showing increased values in both compartments, either in serum or RBC.

**Table 2:** Overview on p-values on day of admission (day 1) to intensive care in samples obtained from COVID-19 patients either from serum or RBC with respect to ceramide concentration differing in chain length of acylated fatty acid 16:0 to 24:1.

		16:0	18:0	20:0	22:0	24:0	24:1
**HC vs. COVID-19**	RBC	0.001	< 0.0005	0.004	0.004	< 0.05	0.007
	serum	0.007	0.001	< 0.0003	0.001	0.028	0.001

Statistical analysis was performed using Mann-Whitney U-Test and p-values <0.05 were considered to be significant.

**Table 3:** Comparing concentration of ceramides differing in chain length of acylated fatty acid 16:0 to 24:1.

RBC [nmol/mL]		16:0	18:0	20:0	22:0	24:0	24:1
**COVID-19**	median	3.42	1.10	0.33	2.00	1.82	6.47
	Q1/Q3	2,98/4,44	0,83/1,37	0,26/0,40	1,76/2,59	1,44/2,42	5,49/8,05
**Healthy controls**	median	1.36	0.43	0.20	1.22	1.31	3.43
	Q1/Q3	1,36/1,38	0,42/0,47	0,12/0,20	0,97/1,34	1,18/1,46	3,01/3,60
**Serum [nmol/mL]**		**16:0**	**18:0**	**20:0**	**22:0**	**24:0**	**24:1**
**COVID-19**	median	1.41	0.39	0.14	0.89	0.78	1.09
	Q1/Q3	1,16/1,67	0,25/0,48	0,12/0,19	0,76/1,08	0,67/0,95	1,01/1,41
**Healthy controls**	median	0.89	0.15	0.05	0.37	0.60	0.52
	Q1/Q3	0,77/1,03	0,10/0,16	0,05/0,06	0,33/0,47	0,48/0,61	0,50/0,54

Given are the median values [nmol/mL] and the interquartile range Q1/Q3 either from serum or RBC samples. Data for statistical comparison are given in **Table 2**.

### Activity of Circulating Sphingomyelinase as Potential Source of Deranged Ceramide Profiles

**Figure 4:**
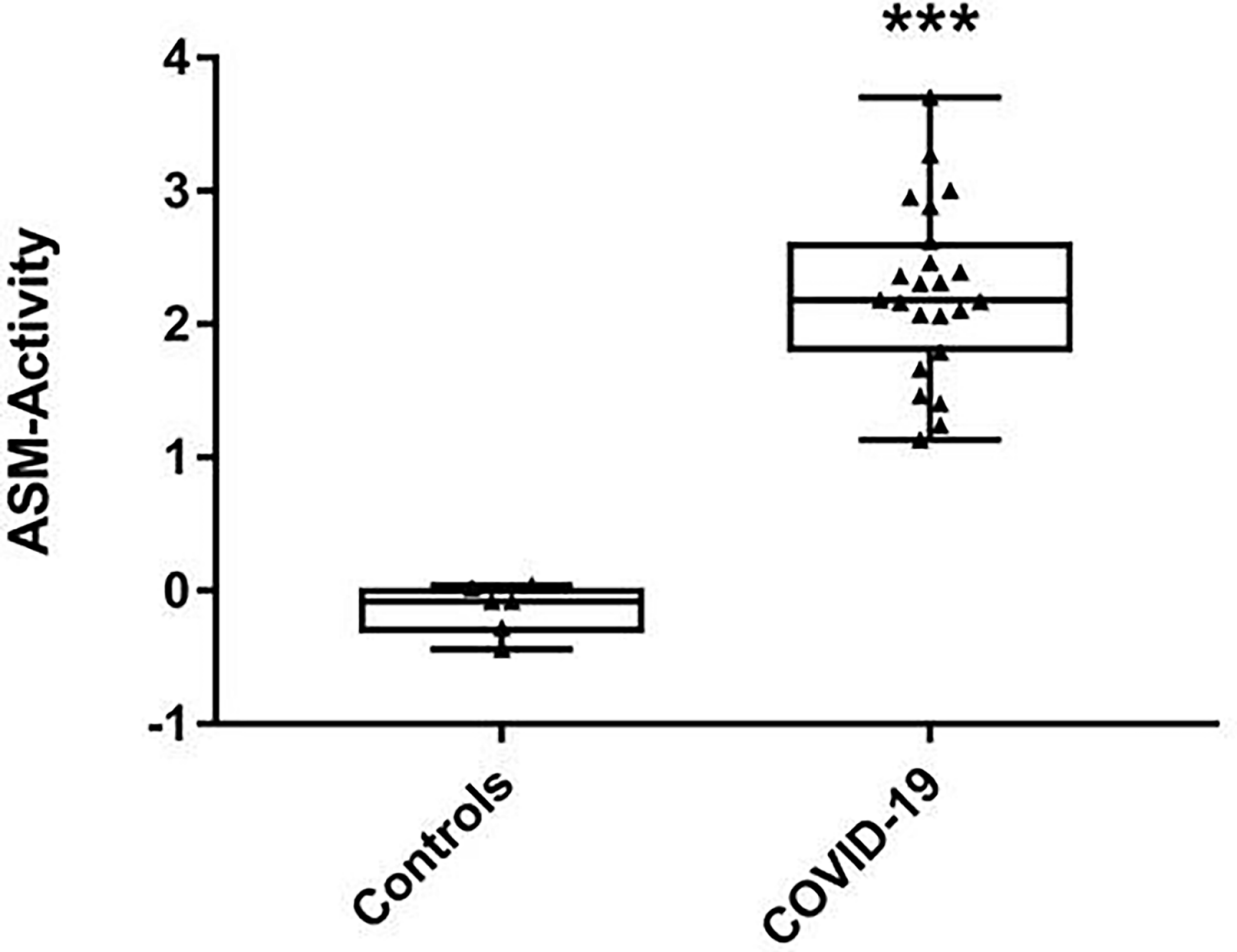
Activity levels of acid sphingomyelinase (ASM) in serum samples. Shown are medians with interquartile ranges of ASM activity levels from 23 severe COVID-19 patients and six volunteers as healthy controls, measured in separate aliquots used for ceramide profiling. Statistical significance was tested using the Mann-Whitney U-test; statistical difference is indicated by asterisks: ***p < 0.0001.

Considering the increase of all investigated ceramide specimen in COVID-19 patients in serum and RBC, we then tested whether the corresponding stress associated enzyme ASM - converting sphingomyelin to ceramide - was more abundant and/or more active in COVID-19 patients. Indeed, in serum of COVID-19 patients the enzyme activity was markedly increased: median 4.525 (Q25% 3.827; Q75% 5.832) nmol/(mL x h) as opposed to 0.948 nmol/(mL x h) (Q25% 0.857; Q75% 0.999) in healthy controls and this effect was statistically significant (**Figure 4**) reflecting an inflammation-driven sphingolipid reprogramming. In patients with unfavorable outcome (n=7), the highly increased activity levels remained nearly unchanged, but in patients with recovery (n=16), we observed decreasing values (data not shown).

### Association of ASM-Activity With Clinical Severity by Correlational Analyses

Correlating ASM activity with clinical parameters at day of ICU admission revealed a clear association of ASM activity and severity of COVID-19 (**Table 4**). Most prominently, we found that the concentration of high density lipoprotein (HDL) negatively correlated with ASM-activity, followed by biomarkers of metabolic dysfunction such as base excess, concentration of lactate and hydrogen carbonate. Further, the concentration of long chain sphingomyelin from serum constituents (lipoproteins) was found to be negatively correlated with ASM-activity. A positive correlation was found for plasma magnesium concentration, alanine aminotransferase (ALT), total bilirubin, ferritin and lactate dehydrogenase.

**Table 4:** Correlational analysis of ASM-activity with clinical and laboratory data.

	rho	CI	p-value	Pairs
High density lipoprotein	-0.6379	-0,8466 to -0,2592	0.0025	20
Base Excess art.	-0.5721	-0,8012 to -0,1969	0.0043	23
Base Excess ven.	-0.5003	-0,7669 to -0,08661	0.0177	22
C26-SM (Serum)	-0.5310	-0,7790 to -0,1394	0.0091	23
Lactate ven.	0.5380	0,1375 to 0,7873	0.0098	22
Lactate art.	0.5218	0,1269 to 0,7739	0.0107	23
HCO_3_ stand. art.	-0.5928	-0,8459 to -0,1216	0.0173	16
HCO_3_ akt. art.	-0.4550	-0,7363 to -0,03981	0.0291	23
HCO_3_, stand. ven.	-0.5097	-0,8011 to -0,02305	0.0383	17
pH(T) ven.	-0.5012	-0,7673 to -0,08772	0.0175	22
pH ven.	-0.4825	-0,7570 to -0,06326	0.0229	22
Mg	0.5493	0,09609 to 0,8139	0.0182	18
Alanine aspartat transferase	0.4955	0,08016 to 0,7642	0.0190	22
total Bilirubin	0.4464	0,02901 to 0,7313	0.0327	23
Ferritin	0.4758	0,01312 to 0,7707	0.0395	19
Lactate dehydrogenase	0.4631	-0,003281 to 0,7640	0.0459	19

Given are the rho-coefficient of correlation (Spearman), the interval of 95% confidence (CI), the absolute p-value (two tailed) and the number of available data for pairwise analysis. C26-SM sphingomyelin with an acylated fatty acid of 26 carbon atoms, respectively.

## Discussion

In this observational trial, activation of plasma circulating sphingomyelinase in response to infection was found in COVID-19 patients undergoing intensive care treatment resulting in an increase of ceramide isoforms generated from inert membrane constituent sphingomyelin in RBC.

### Release of ASM by an Imbalanced Repair Mechanism

It might be speculated, that the origin of the circulating enzyme activity might be ascribed to loss of integrity of epithelial tissues in affected lungs, of which severe damage of alveolae beyond remodeling of parenchyma is manifest along histopathological findings of the disease (40, 41). Exocytosis of ASM is a common and rather protective mechanism for rapid membrane resealing and restoration of its integrity (18, 42). The appearance of ASM in circulation is a sensitive, but unspecific event in patients with severe inflammation resulting from infection such as polymicrobial sepsis (19, 20), pneumonia (21), radiation therapy (43) or chronic inflammation (44). In community-acquired pneumonia patients, Arshad et al. recently reported a nearly threefold increase of plasma ASM activity, its close correlation with severity markers (C-reactive protein, procalcitonin), a concomitant increase of SMPD1-expression in circulating white blood cells (twofold), and, most interestingly, a derangement of plasma ceramide profile (21), which is very similar to that we found in our study. There are several reports on correlations between plasma ceramide concentration and unfavorable outcome of critically ill patients (45, 46), especially in the lungs (47). On a molecular level, ceramides have been reported to activate inflammatory pathways in several abnormal physiological circumstances involving insulin resistance, mitochondrial dysfunction and endoplasmatic reticulum stress (48–51), which are attributed to worsen the clinical condition of COVID-19 patients (52–54). Despite the fact, that we are only presenting ceramide data from accessible compartments from these patients (serum and RBC), a close similarity of membrane bound ceramides in bodies’ tissues with that we analyzed is persuasive, since the hydrolyzing capacity is restricted to ASM in these conditions.

### Ceramide Functioning During Development of Long-Term Sequelae of COVID-19

As a long-term sequelae of COVID-19, lung fibrosis is characterized by deterioration of organ function and subsequent respiratory failure (55). Since high quality data regarding long-term clinical outcomes from COVID-19 survivors are still unavailable, predictions for long-term outcome thereof are speculative at best, but it is well known that lung fibrosis as a result of other diseases closely correlates with poor prognosis (56). The underlying, irreversible process is driven by sometimes excessive release of pro-fibrotic factors (56), especially TGF-β from injured lung tissue, turning a well-controlled healing response into a pathogenic fibrotic response (56, 57). In follow-up chest imaging from severe courses, the presence of intestitial thickening, irregular interface and parenchymal bands have been suggested as predictors of COVID-19 pulmonary fibrosis (58). Generation and accumulation of ceramides were identified as pacemakers in pathogenesis of pulmonary fibrosis in cystic fibrosis in mice and men (59–61). As a consequence, inhibition of ASM resulted in normalization of pulmonary ceramide levels, inflammation and bacterial infection (62–64).

These and also previous observations from our group demonstrated that an increase of ASM-activity, ceramide formation, TGF-β circulation and - ultimately - liver fibrosis in a mouse model of polymicrobial sepsis might be abrogated by ASM-inhibition (65). These findings also support the concept that activation of the sphingomyelinase-ceramide-pathway is a universal response mechanism during host response. Furthermore, inhibition of the enzyme might have both, short- and long-term beneficial effects during the course of the disease. Especially, in high risk patients anti-fibrotic therapy is a matter of debate (66). Strikingly, data from a small series of independent retrospective studies, clearly attest a benefit for patients undergoing anti-depressive therapy precedent to hospitalization due to COVID-19 (67–70). There is an anticipation that anti-depressive drugs as **f**unctional **i**nhibitors of **s**phingo**m**yelin**a**se (FIASMA) (71) exhibit anti-infective properties in epithelial cell with respect to SARS-CoV-2 (24, 72). Hoertel et al. showed a significant association between the prehospital use of antidepressants with subsequent inhibition of ASM and reduced likelihood of intubation or risk of death due to SARS-CoV-2 complication (67). Notably in older adults, the short-term use of FIASMA is generally well tolerated (73, 74). A trend for beneficial effects of FIASMA with respect to in-hospital mortality rate (potentially marked by older age and higher prevalence of comorbidities) was superimposed by co-medication with amlodipine (70). The later drug also exerts inhibitory capacity to ASM, but also antiviral effects *in-vitro* (75, 76) might be exaggerated by the Ca^++^-modulating mechanism (77). In line with these results, there are similar data from small studies demonstrating a lower mortality rate after treatment with nifedipine or amlodipine (75, 78). A recent update on the potential role of either a chronic expose to FIASMA or as an interventional measure following hospitalization due to SARS-CoV-2 infection underlines the interest to evaluate these drugs as off-label therapy in SARS-CoV-2 infection (79). Interestingly, recent reports demonstrated a synergistic effect of the FIASMA fluoxetine and the direct anti-viral agent remdesivir and its metabolite in an *in-vitro*-model of polarized Calu-3-cells: super-additive effectiveness highlighted key advantages of a combined approach against the propagation of the viral pathogen as well as maintenance of endosomal lipid balance for entry processing into the host at low concentrations minimizing potential adverse effects of the drugs (80, 81).

### Ceramide Function in RBC

RBC play an important role in oxygen transport and supply as well as they are fulfilling a plethora of metabolic activities. Within this cell population, ASM-triggered ceramide generation contributes to Ca^++^-sensitivity resulting in the release of extracellular vesicles by shaping the membrane’s curvature (82) and induction of eryptosis, the suicidal death of RBC (83, 84). Also, ASM induced ceramide generation changes the biophysical properties (85) resulting in an increase of rigidity in membranes (86, 87) impairing the function of RBC. This is in line with observations from anemic COVID-19 patients, exhibiting RBC shape abnormalities and morphological changes leading to a spherocyte shape, which are all characterized by loss of elastic properties (88). After recovery, blood smear showed unremarkable morphology (88). The same is true with respect to formation and release of extracellular vesicles, since ceramide generation is pace-making as shown by studies using inhibitors of both isoforms of sphingomyelinase (*i.e.* GW4869, imipramine) (89). Beside the fact of increased levels of circulating extracellular vesicles that may drive thrombosis in patients undergoing COVID-19 (90), to the best of our knowledge, there are no clinical observations of RBC-borne vesicles as yet.

Data from our correlational analyses support the hypothesis that ASM activity is either a mediator or marker of a severe clinical course in our patient cohort. It is well described that patients with decreased HDL-levels are at an increased risk to develop a severe disease course compared to patients with high HDL levels (91). Here, low HDL values are found to be decreased in COVID-19 patients (**Table 1**) and are associated with increased ASM activity (**Table 4**). Most interestingly, ASM activity is found to be associated with biomarkers of metabolic dysfunction such as hydrogen carbonate, base excess and lactate concentration, of which the latter one was found in our patients in normal range (**Table 1**). Unlike polymicrobial sepsis, in COVID-19 lactate levels are usually normal despite severe pneumonia or manifest ARDS without any prognostic value with respect to outcome (27, 92). In our study, ASM-activity and lactate levels are highly correlating at the day of admission to the intensive care unit. It is unclear, whether low levels of lactate are caused by increased consumption by lactate dehydrogenase (93), which is also found to be associated with ASM-activity. Increased values of lactate dehydrogenase activity (LDH) at hospitalization are positively associated with mortality (93). Considering the parallel alteration of ASM and LDH, one might speculate that both enzymes might be released probably from injured heart and lung tissue (27). Therefore, it might be interesting to determine the enzymatic activity of proteins in bronchoalveolar fluid, where a similar change might be expected. Furthermore ASM activity is positively associated with marker of impaired liver function such as ALT and bilirubin, which are both (beyond LDH) previously described as screening prognosticators of severe courses at early stages of the disease (94). The same is true with respect to ferritin as a surrogate for hyper-immune responsiveness, since baseline levels at ICU-admission are increased (**Table 1**) (95). Established biomarkers such as procalcitonin (p= 0.065) or SOFA-values (0.138) failed to reach the level of significance due to the small cohort of patients. Interestingly, there is no association of ASM activity with values of troponin I in patients as a surrogate for damage of myocardial tissue (p=0.3365), thus the serum abundant ASM activity in COVID-19 is proposed to be released from epithelial tissue of the affected lungs as previously described in asthma (96, 97) and acute respiratory syncytial virus bronchiolitis (98).

### Limitations

Regardless of these promising new results, this study is also confronted with particular limitations. Our study was carried out at a single center with a quite limited number of patients, all of them admitted to the intensive care unit. Nevertheless, we are convinced, that the cohort is sufficiently powered for the presented results. However, the size of the cohort does not allow for the analysis of distinct subsets such as outcome, need for ventilation, or any prediction of the consequences of high/low levels of sphingomyelinase from circulation or in RBC. Furthermore, we cannot exclude that our results are biased by sample size or (anti-depressant) treatment strategies. We agree that data about prehospital treatment with FIASMAs would strengthen our results but due to the observational design and the confirmative character of the study of an unknown hypothesis we are not able to provide those data. Thus, also considering the small group size, a comparative analysis of our patients with respect to pretreatment with FIASMAs might over expand the interpretation of our results.

We measured sphingomyelinase activity and subsequent derangement of ceramide profile in serum/RBC and correlated the measured values with clinical and laboratory parameters. But our observations cannot explain cause-consequences at the end, especially whether the increase of enzyme activity is an epiphenomenon of or a reason for deterioration with subsequent need for intensive care treatment. Our study, without external validation, was primarily not designed to assess long-term outcomes and was therefore not feasible to screen for prognostic biomarkers for long-term sequelae. Nonetheless, our data highlight a potentially crucial signaling pathway in COVID-19 patients that warrants further investigations. Notably, the activation of circulating sphingomyelinase (ASM) and subsequent ceramide generation during host response in these patients provide a promising approach for functional inhibition by FDA-approved drugs to control resulting organ dysfunction to help the body maintaining homeostasis. Larger multicenter, interventional trials are now needed to test the potential benefit of an inhibitory strategy in critically ill patients with COVID-19.

### Conclusions

The data from our study close the gap between retrospective observations by presenting a potential mechanism of ASM release and action in COVID-19 patients. Keeping the paucity of proven host-directed therapies in mind, the low level of evidence of the majority of all running trials (14, 99) and due to the fact that a panel of FDA-approved drugs with low risk of adverse effects is awaiting consequent investigation, the potential usefulness of anti-depressants in patients with COVID-19 taking FIASMA for other indications with now known guiding principles should be prioritized for RCT and can minimize the risk of being exposed to novel, potentially harmful of ineffective compounds or compounds with unknown mode of action. As also recommended by others, the results support the continuation of FIASMA medication in these patients (69). Moreover, the fact of pretreatment with FIASMA should be considered while interpreting the results from hundreds of running clinical trials (17). Double-blind controlled randomized clinical trials of antidepressive medication with FIASMA for transient inhibition of ASM in COVID-19 are of great interest to investigate how the ASM/ceramide-pathway affects disease severity, organ damage and improvement of clinical course.

## Data Availability Statement

The raw data supporting the conclusions of this article will be made available by the authors, without undue reservation.

## Ethics Statement

The local ethics board at UMG approved inclusion of all ICU patients (reference 15/4/19Ü). The patients/participants provided their written informed consent to participate in this study.

## Author Contributions

Conceptualization: MW and RC. Methodology: MA, MG, RC, and MW. Formal analysis: MA, MG, and RC. Investigation: MA, MW, MG, and RC. Clinical characterization: MW, OM, BT, SP, KM, and HH-W. Resources: MG and MB. Writing – Original Draft: MA, MG, MW, and RC. Writing – Review & Editing: all authors. Visualization: MA, RC, MG, and MW. Supervision: RC and MW. Project Administration: MW, RC, and MG. Funding Acquisition: MG and KM. All authors contributed to the article and approved the submitted version.

## Funding

The study was supported by institutional grants to RC, MG, and MW. We also acknowledge support by the German Research Foundation and the Open Access Publication Fund of the Thueringer Universitaets- und Landesbibliothek Jena Projekt-Nr. 433052568. MW received unrestricted funding from SARTORIUS. The funder was not involved in the study design, collection, analysis, interpretation of data, the writing of this article or the decision to submit it for publication.

## Conflict of Interest

The authors declare that the research was conducted in the absence of any commercial or financial relationships that could be construed as a potential conflict of interest.

## Publisher’s Note

All claims expressed in this article are solely those of the authors and do not necessarily represent those of their affiliated organizations, or those of the publisher, the editors and the reviewers. Any product that may be evaluated in this article, or claim that may be made by its manufacturer, is not guaranteed or endorsed by the publisher.

## Footnotes

1 Winkler MS, Claus RA, Schilder M, Hoffmann-Winkler H, Coldewey SM, Moerer OM, Meinsner K, Bauer M, Pöhlmann S, Gräler MH: Erythrocytes increase endogeneous sphingosine-1-phosphate (S1P) levels as an adaptive response to SARS-CoV-2 infection. *Clin Sci (Lond)*. 2021 Dec 8:CS20210666.
