# Supplementary material for: Activation of Sphingomyelinase-Ceramide-Pathway in COVID-19 Purposes Its Inhibition for Therapeutic Strategies
Source: Front Immunol. 2021 Dec 20;12:784989. doi: 10.3389/fimmu.2021.784989 (PMC8721106; doi:10.3389/fimmu.2021.784989)
Supplement: Supplementary file 1 [file DataSheet_1.docx]

**Method Supplement**

**Activation of Sphingomyelinase-Ceramide-Pathway in COVID-19
Repurposes its Inhibition into Therapeutic Strategies**

Murad Abusukhun^1,2^*, Martin S. Winkler^3^*, Stefan Pöhlmann^4,5^,
Onnen Moerer^3^, Konrad Meissner^3^, Björn Tampe^6^, Heike Hofmann-Winkler^4^,
Michael Bauer^1,7^, Markus H. Gräler^1,2,7^, Ralf A. Claus^1,2 †^

** both first authors contributed equally to this study.*

*Study population –* The first 23 COVID-19 patients treated in the intensive care unit (ICU) of the Department of Anesthesiology at Göttingen University Medical Centre (UMG) from March 2020 to May 2020 were enrolled into this study. The local ethics board at the University Medical Center of Göttingen approved inclusion of all ICU patients (reference 25/4/19Ü). Informed consent has been obtained from patients or their legal representatives before inclusion. Data from this report are achieved from a re-analysis of samples firstly reported in 2021^[[1]](#footnote-1)^.

*Clinical evaluations and assays -* SOFA scores were calculated on admission according to the published guidelines [1]. Within the first 24h after inclusion, plasma and serum samples were taken to measure ceramide profile and circulating sphingomyelinase activity. The Institute of Clinical Chemistry and Laboratory Medicine at UMG measured the concentration of albumin together with all other inflammatory markers. Leukocyte-free RBC were harvested from separate samples by density gradient centrifugation, washed and stored in plasma-free conditions at 4 °C.

*Extraction and quantification of ceramides -* Ceramide measurements were performed according to an established protocol using liquid chromatography coupled to triple-quadrupole mass spectrometry (LC-MS/MS) [2]. Samples (20 µL) from plasma and serum as well as 20 µL pelleted RBC (1 x 10E7 cells) were transferred to glass centrifuge tubes and proteins were precipitated by addition of 10X volume of methanol, supplemented with appropriate internal standard solutions (30 pmol C15-Ceramide (d18:1) and deuterated d31:C16-ceramide per sample for regular analyses, Avanti Polar Lipids, Alabaster, AL, USA). After incubation over night at -80°C and centrifugation (14,000 rcf for 5 min), supernatants were vacuum-dried at 60 °C for 50 min using a vacuum concentrator. The extracted lipids were dissolved in 100 μl methanol/chloroform (4:1, v/v) and stored at -20 °C. Detection was performed with the QTrap triple-quadrupole mass spectrometer (Sciex, Darmstadt, Germany) interfaced with the 1100 series chromatograph (Agilent, Frankfurt, Germany) and the Hitachi Elite LaChrom column oven and autosampler (VWR, Darmstadt, Germany). Positive electrospray ionization (ESI) LC/MS/MS analysis was used for detection of sphingomyelins, positive atmospheric pressure chemical ionization (APCI) for ceramides. Multiple reaction monitoring (MRM) transitions m/z were as follows:

|  | **Sphingomyelins (d18:1)  > 184.10** | |  | **Ceramides (d18.1) > 264.30** | | **comment** |
| --- | --- | --- | --- | --- | --- | --- |
| Fatty acid | :0 | :1 |  | :0 | :1 |  |
| 12 | 647.7 | ./. |  | 482.6 | ./. |  |
| 14 | 675.7 | ./. |  | 510.7 | ./. |  |
| 15 | ./. | ./. |  | 524.4 | ./. | IS (APCI mode) |
| 16 | 703.8 | ./. |  | 538.7 | ./. |  |
| 17 | 717.5 | ./. |  | 552.4 | ./. | IS (ESI mode)  Substrate/product |
| 18 | 731.8 | 729.8 |  | 566.7 | 564.7 |  |
| 20 | 759.9 | ./. |  | 594.7 | ./. |  |
| 22 | 787.9 | ./. |  | 622.8 | ./. |  |
| 24 | 815.9 | 813.9 |  | 650.9 | 648.9 |  |
| 26 | 843.9 | 841.9 |  | ./. | ./. |  |

Liquid chromatographic resolution of all analytes was achieved using a 2 x 60 mm MultoHigh C18 reversed phase column with 3 μm particle size (CS-Chromatographie Service, Langerwehe, Germany). The column was equilibrated with 10% methanol and 90% of 1% formic acid in H_2_O for 5 min, followed by sample injection and 15 min elution with 100% methanol with a flow rate of 300 μl/min. Standard curves were generated by adding increasing concentrations of ceramide to 100 pmol of the internal standard C15-ceramides. Linearity of the standard curves and correlation coefficients were obtained by linear regression analyses (r^2^ > 0.99). Data analyzes were performed using Analyst 1.6 (Sciex).

*Sphingomyelinase activity assay* – Serum samples, 10 µL each; from healthy controls: 20 µL) were dissolved with 70 µL (healthy controls: 60 µL) reaction puffer (sodium acetate 100 mM, zinc acetate 160 µM, 0.01 % NP40, pH 5.0) and supplemented with 20 µL of substrate solution (200 µM SM-d18:1/17:0, 0.01% NP40, supplemented with 15 µM Cer-d18.1/15:0 as internal standard). Samples were incubated for four hours at 37 °C in a water bath. Reaction was stopped by addition of 1 mL methanol. Samples were vigorously vortexed for 10 min. After overnight precipitation of proteins, supernatant were separated by centrifugation at 4 °C for 10 min at 3,500 rcf, which were transferred into a new glass centrifuge tube. After evaporation (50 °C for 120 min) using a vacuum concentrator, the extracted lipids were dissolved in 100.0 μL methanol/chloroform (4:1, v/v) and stored at -20 °C. Detection of C17-Cer (d18:1) for the conversion from C17-SM (d18:1) was performed with the API2000 triple-quadrupole mass spectrometer as described above.

*Statistical analysis -* The primary variables were ceramide isoforms (n=6) in plasma, serum and RBC, and C17-Cer(d18:1) as product of sphingomyelinase activity. Outliers were identified according to the ROUT method with the desired maximum false discovery rate Q set to 1 % [3]. Differences between two groups were tested for significance using the unpaired Student’s t-test with nonparametric correction using the Mann-Whitney U-test. A p-value <0.05 was considered to be significant. Statistical analyses were performed using Graph Pad Prism 7.0a, April 2016 (La Jolla, CA, USA) as well as IBM SPSS Statistics 27. Graphical illustration of variations and principal component analyses were performed using metaboanalyst platform (5.0 [4])

**References**

1. Winkler, M.S.; Kluge, S.; Holzmann, M.; Moritz, E.; Robbe, L.; Bauer, A.; Zahrte, C.; Priefler, M.; Schwedhelm, E.; Boger, R.H., et al. Markers of nitric oxide are associated with sepsis severity: an observational study. *Crit Care* **2017**, *21*, 189, doi:10.1186/s13054-017-1782-2.

2. Bode, C.; Graler, M.H. Quantification of sphingosine-1-phosphate and related sphingolipids by liquid chromatography coupled to tandem mass spectrometry. *Methods Mol Biol* **2012**, *874*, 33-44, doi:10.1007/978-1-61779-800-9_3.

3. Motulsky, H.J.; Brown, R.E. Detecting outliers when fitting data with nonlinear regression - a new method based on robust nonlinear regression and the false discovery rate. *BMC Bioinformatics* **2006**, *7*, 123, doi:10.1186/1471-2105-7-123.

4. Pang, Z.; Chong, J.; Zhou, G.; de Lima Morais, D.A.; Chang, L.; Barrette, M.; Gauthier, C.; Jacques, P.E.; Li, S.; Xia, J. MetaboAnalyst 5.0: narrowing the gap between raw spectra and functional insights. *Nucleic Acids Res* **2021**, *49*, W388-W396, doi:10.1093/nar/gkab382.

1. Winkler MS, Claus RA, Schilder M, Hoffmann-Winkler H, Coldewey SM, Moerer OM, Meinsner K, Bauer M, Pöhlmann S, Gräler MH: Erythrocytes increase endogeneous sphingosine-1-phosphate (S1P) levels as an adaptive response to SARS-CoV-2 infection. *Cell. Sci.*, under revison. [↑](#footnote-ref-1)
